# The Effects of Sulfonated Poly(ether ether ketone) Ion Exchange Preparation Conditions on Membrane Properties

**DOI:** 10.3390/membranes3030182

**Published:** 2013-08-13

**Authors:** Rebecca S. L. Yee, Kaisong Zhang, Bradley P. Ladewig

**Affiliations:** 1 Department of Chemical Engineering, Monash University, Clayton VIC 3800, Australia; E-Mail: rebecca.yee@monash.edu; 2 Institute of Urban Environments, Chinese Academy of Science, 1799 Jimei Road, Xiamen 361021, China; E-Mail: kszhang@iue.ac.cn

**Keywords:** sulfonated poly(ether ether ketone), SPEEK, bioelectrochemical systems, cation exchange membrane, low cost, ion exchange

## Abstract

A low cost cation exchange membrane to be used in a specific bioelectrochemical system has been developed using poly(ether ether ketone) (PEEK). This material is presented as an alternative to current commercial ion exchange membranes that have been primarily designed for fuel cell applications. To increase the hydrophilicity and ion transport of the PEEK material, charged groups are introduced through sulfonation. The effect of sulfonation and casting conditions on membrane performance has been systematically determined by producing a series of membranes synthesized over an array of reaction and casting conditions. Optimal reaction and casting conditions for producing SPEEK ion exchange membranes with appropriate performance characteristics have been established by this uniquely systematic experimental series. Membrane materials were characterized by ion exchange capacity, water uptake, swelling, potential difference and NMR analysis. Testing this extensive membranes series established that the most appropriate sulfonation conditions were 60 °C for 6 h. For mechanical stability and ease of handling, SPEEK membranes cast from solvent casting concentrations of 15%–25% with a resulting thickness of 30–50 µm were also found to be most suitable from the series of tested casting conditions. Drying conditions did not have any apparent impact on the measured parameters in this study. The conductivity of SPEEK membranes was found to be in the range of 10^−3^ S cm^−1^, which is suitable for use as a low cost membrane in the intended bioelectrochemical systems.

## 1. Introduction

Sulfonated PEEK membranes have been extensively developed for use in fuel cells. These materials can also be used in electrochemical systems such as microbial bioreactors. In this paper, we produce a suitable PEM for use in a specific bioelectrochemical system (BES) that has been developed in other literature [1]. Our work will be used in relation to this unique BES with the aim of recovering valuable chemicals from industrial wastewater. This BES requires a selective cation exchange membrane that can operate in caustic conditions with high current densities. 

Currently, most membranes have been developed with microbial fuel cell conditions in mind [2], with few membranes produced specifically for BES operation. Until recently, materials and designs employed for pilot scale BESs employed the same membranes as used for ion exchange fuel cells, such as Nafion^®^ 117. However, the high cost of perfluorinated Nafion membranes makes them unsuitable for low profit margin wastewater treatment systems. 

Poly(ether ether ketone) (PEEK) is a high performance engineering thermoplastic that has good solvent resistance, high thermo-oxidative stability and excellent mechanical properties. The sulfonated derivatives of this polymer offer low cost alternatives to Nafion^®^ membranes [3]. The aromatic backbone helps maintain thermal and mechanical stability, as well as allowing for chemical modification such as through simple electrophilic substitution by sulfonation. A sulfonating agent can incorporate sulfonated groups onto the polymer chains, either directly or by polymerizing functionalized monomers. The degree of sulfonation can be controlled by reaction time and temperature [4].

To increase the hydrophilicity of PEEK materials, charged groups are introduced using sulfuric acid into the polymer chains to make them ion-exchangeable. Sulfonation aids in the transport of cations and increases the hydrophilicity of the polymer. The sulfonate groups (–SO_3_–) can be introduced into the PEEK polymer chain using several methods. Sulfonation can be done pre- or post-polymerization. Pre-sulfonation process offers better control of sulfonation degree by being able to vary the ratio of modified monomers in the final polymer and has been examined for producing SPEEK membranes for fuel cells [5,6,7,8,9,10]. However, this method of modification is too complex and impractical for large-scale commercial use. Post-sulfonation is more widely used due to its simplicity and low production costs [6,11,12,13,14]. 

Much research has concentrated on reducing methanol crossover for fuel cell applications [15,16]. However, few works have focused on using PEEK as a polymer matrix for bioelectrochemical systems requiring lower ion exchange capacity membranes that allows for lower cost materials to be used. For such technologies, ion exchange membranes with conductivities in the range of 3–5 mS/cm and ion exchange capacities in the range of 1–2 mol/kg are desirable [11]. There have also been limited publications focusing on the relationship between SPEEK preparation conditions involving synthesis and membrane formation, and the resulting membrane structure and properties. 

The sulfonation of PEEK is a second order electrophilic reaction used to add charged groups into the polymer chains to make them ion-exchangeable. The addition of the charged groups depends on the substituents present in the ring [17,18]. Electron-donating substituents will favor reaction whereas electron-withdrawing groups will not. In PEEK, the hydroquinone unit between the ether bridges can be sulfonated under relatively mild conditions [4]. Substitution takes place preferentially on the aromatic ring between two ether (–O–) links. At higher temperatures, substitution of the other aromatic rings is also possible.

Several methods for the sulfonation of SPEEK have been previously described in literature [16]. PEEK can be functionalized by sulfonation where the degree of sulfonation is controlled by reaction time and temperature. However, the sulfonation conditions used in previous SPEEK production have not previously been optimized by any systematic approach. Most previous research has selected arbitrary conditions for SPEEK membrane synthesis. This paper tests an extensive array of membranes produced at various reaction times and temperatures to establish an optimal combination of variables to synthesize appropriate membrane for future large-scale development. Membrane casting and preparation conditions can also affect the performance of produced SPEEK membranes. Klaysom (2011) has previously examined the effects of drying on the membrane structure and subsequent performance characteristics for sulfonated PES [19]. The aim of this paper is to determine the effects of the sulfonation, casting and drying conditions on SPEEK membrane performance through the systematic study of a series of membranes produced over a range of influential variables. Little previous literature has looked at such systematic characterization for determining optimal conditions for large-scale SPEEK ion exchange membrane production. In particular, these production conditions will be used for future development of SPEEK membranes implemented in the novel BES described previously [1]. 

These results will be used to develop better criteria to design new polymer membranes with high performance for bioelectrochemical systems.

## 2. Results and Discussion

2.1. ^1^H-NMR Structural Analysis

**Figure 1:**
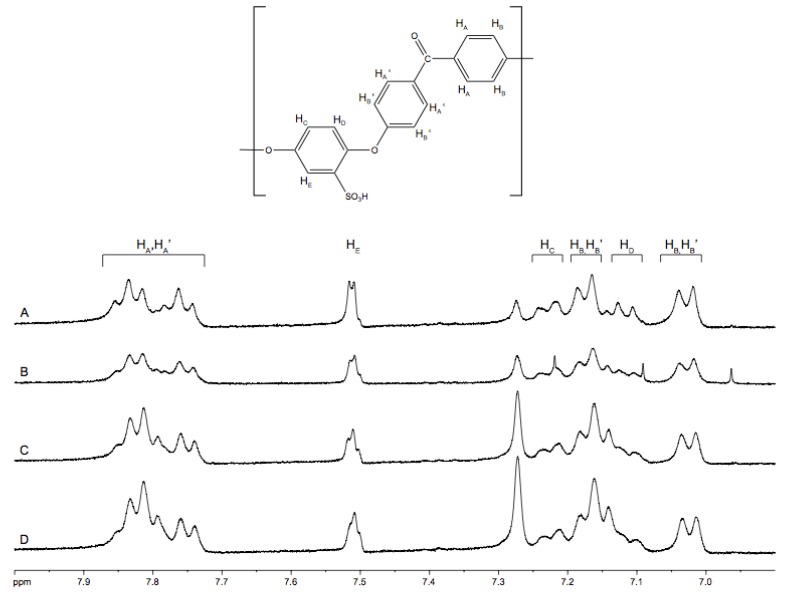
NMR analysis of sulfonated poly(ether ether ketone) (SPEEK) membranes produced at various sulfonation temperatures and reaction times; (**A**) 60 °C, 6 h; (**B**) 50 °C, 6 h; (**C**) 60 °C, 2 h; (**D**) 50 °C, 2 h.

The structure of the sulfonated SPEEK was determined by ^1^H-NMR analysis. The spectra and nomenclature of the aromatic protons for the SPEEK repeat unit is shown in Figure 1.

The presence of the sulfuric acid group causes a down-field shift of the hydrogen, H_E_, to 7.50 ppm. The doublets at 7.15 and 7.25 can be assigned to H_C_ and H_D_ on the hydroquinone ring. When more sulfonic groups attached to the aromatic ring in the PEEK repeat unit, the intensity of the signal was enhanced as expected. In particular, the peak at 7.27 grows noticeably smaller as the degree of sulfonation increases. 

The intensity of the H_E_ signal provides an estimate of the SO_3_H group content. The ratio of the peak area of the distinct H_E_ (AH_E_) and the integrated peak area of the signals corresponding to all the other aromatic hydrogens (AH_A,A′,B,B′,C,D_) can be expressed as:

n/(12 − 2n) = AH_E_/ΣAH_A,A′,B,B′,C,D_ (1)

where AH_E_ is the peak area of the H_E_ signal, and ∑AH_A,A′,B,B′,C,D_ is the sum of the peak area of the signals corresponding to all the other aromatic hydrogens. The degree of sulfonation (DS) can be obtained from DS = n × 100%. 

### 2.2. Degree of Sulfonation

The DS is defined as the percentage of repeat PEEK units that have been sulfonated. A higher degree of sulfonation indicates that more repeat units have been sulfonated. This can be determined using either the IEC found by the titration method or by ^1^H-NMR spectroscopy. 

Using the titration method to find the IEC, the degree of sulfonation can be calculated to determine the amount of H^+^ cations released from the membrane. By measuring the amount of sulfuric acid consumed in the titration, the molar quantity of –SO_3_H that is present can be determined, where 288.30 is the molecular weight of the PEEK repeat unit and 81.07 is the molecular weight of the –SO_3_H group. The DS can then be found using the following relation.

DS = molar number of PEEK − SO_3_Na unit/(molar number of PEEK − SO_3_Na unit + molar number of PEEK unit).

The degrees of sulfonation found using titration and NMR are shown in Table 1. The results are consistent, indicating accurate measurement of both methods. As expected, the higher sulfonation temperature and longer sulfonation time results in SPEEK membranes with higher sulfonation degrees. These values are within a reasonable range for use as ion exchange membranes in the proposed BESs.

**Table 1:** Degree of sulfonation as determined by ^1^H-NMR and titration.

Sulfonation temperature (°C)	Sulfonation time (h)	Degree of sulfonation—^1^H NMR	Degree of sulfonation—IEC
60	6	0.85	0.87
60	2	0.70	0.71
50	6	0.73	0.65
50	2	0.64	0.67

### 2.3. Influences of Time and Temperature on Degree of Sulfonation

**Figure 2:**
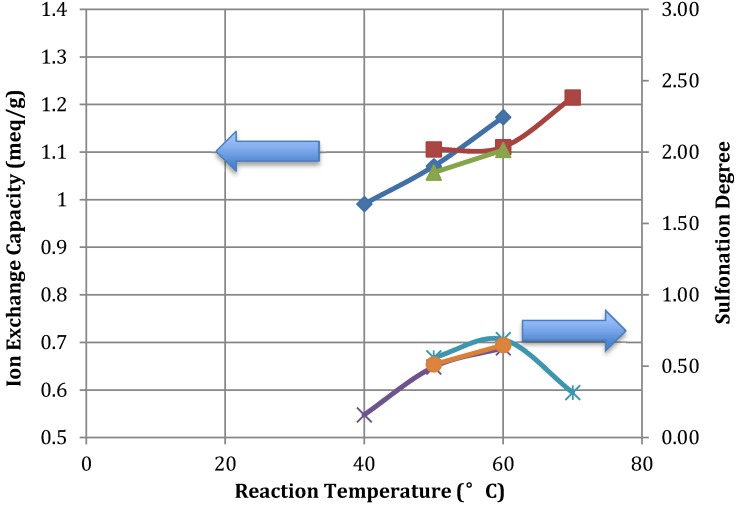
SPEEK IEC and degree of sulfonation (DS) for various reaction temperatures (×, 6 h SD; ○, 3 h SD; *, 2 h SD; diamond, 6 h IEC; ∆, 3 h IEC; □, 2 h IEC).

The sulfonation temperature was found to be the most influential parameter on the degree of sulfonation and IEC, with increased temperatures causing an increase in IEC, water uptake and swelling of the membranes. This behaviour is indicated in Figure 2. This is consistent with literature [15,20,21,22,23,24,25] where the added sulfonate groups provide the formation water-mediated pathways for cation transfer. 

Preliminary investigations found that sulfonation temperatures below 40 °C were too low to effectively sulfonate the PEEK polymer within a reasonable time frame. The produced SPEEK was insoluble in DMAc or other organic solvents at room temperature. Sulfonation times of 24 to 112 h at these temperatures were required in other studies to produce membranes with reasonable IEC values of more than 1.2 mequiv/g [4]. This paper looks to identify potential synthesis methods that can be used for large-scale production of membranes for BESs. As such, at 40 °C, the time required to achieve reasonable IEC values is impractical for commercial manufacturing. 

At temperatures higher than 70 °C, SPEEK was over-sulfonated even after short reaction periods of 1 h. Over-sulfonation leads to excessive swelling and membrane deterioration in water. The SPEEK polymer dissolved rapidly in weak solvents including water, making them impractical for membrane casting.

To achieve maximum sulfonation degree with reasonable mechanical stability, sulfonation temperatures of 50–60 °C were used to produce suitable membranes. Sulfonation temperatures below these values resulted in low IEC while higher temperatures caused membranes to dissolve in water. 

**Figure 3:**
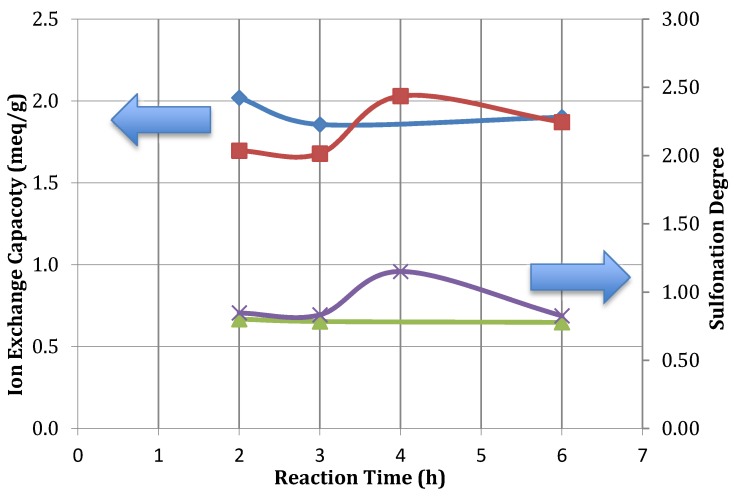
SPEEK IEC and DS various reaction times (○, 50 °C IEC; □, 60 °C IEC; ∆, 50 °C SD; ×, 60 °C SD).

For SPEEK membranes intended for use in bioelectrical systems, an ion exchange capacity of 1–2.5 mequiv/g is desired. Figure 3 show that the achieved IEC is within this range, allowing these membranes to be suitable for the intended systems. 

It was noted from Figure 3 that sulfonation time was not as significant as temperature in affecting the degree of sulfonation. With increased sulfonation time, there was no observable correlation between the sulfonation degree or water uptake values. The degree of sulfonation has been found to depend on reaction time but only over durations of 24 h or more [4,17,18,21]. The scope of this research is focused on potential commercialization that will produce membranes in practical time frames with suitable sulfonation degree. Considering the potential for commercial production, 6 h was considered as an appropriate reaction time. 

### 2.4. Solubility and Swelling

SPEEK membrane properties depend on the concentration of sulfonic groups and the nature of the counter ions within the membrane structure. Sulfonation of the polymer enhances the hydrophilicity and solubility of the membranes [4]. The presence of water molecules in the channels of the microscopic polymer structure significantly affects the ionic conductivity of the resulting membranes due to the increased number of protonic sites on the SO_3_H group, which provides a water-mediated pathway for ion transport [26]. A threshold of water in the membrane matrix is required to maintain ion conductivity. However, excessive water uptake will result in undesirable effects such as membrane swelling, mechanical frailty and lower dimensional stability, leading to poor performance. 

With the aim of implementing these cations exchange membranes in unique BESs for chemical extraction from wastewater, cations will transported across the membrane. Similar studies on the ion transport properties of such membranes have been expressed by Sevada *et al.* (2013) [27]. In the conditions of our intended BES, monovalent cations are desired to be transported across a selective membrane to the cathode side of the system. 

**Figure 4:**
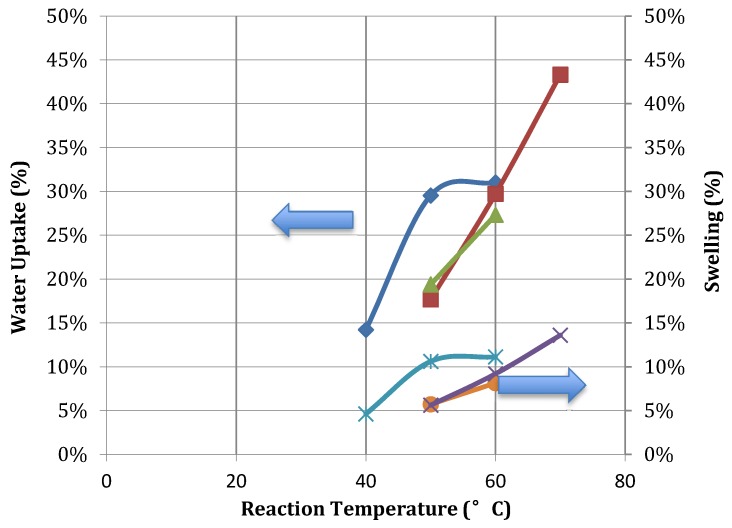
SPEEK membrane water uptake and swelling according to reaction temperature (×, 6 h swelling; ○, 3 h swelling; *, 2 h swelling; diamond, 6 h WU; ∆, 3 h WU; □, 2 h WU).

Figure 4 indicates that both swelling and water uptake increases with sulfonation temperature. A sulfonation degree in the range of 60%–80% was found to successfully enhance hydrophilicity while still maintaining reasonable structural robustness. Water uptake between 20% and 40% was most suitable to allow for high levels of ion conductivity while remaining mechanically stable. At sulfonation degrees lower than 60%, SPEEK was only soluble in strong acids such as sulfuric acid. Above sulfonation degrees of 80%, the SPEEK polymer was soluble in methanol and at 100% sulfonation was soluble in hot water. Table 2 summarizes the solubility of SPEEK produced at varied sulfonation times and temperatures. 

**Table 2:** Solubility of SPEEK produced at various sulfonation temperatures and reaction times.

Time	30 °C	40 °C	50 °C	60 °C	70 °C
1 h	O	O	O	X	X
2 h	O	O	O	X	U
3 h	O	O	X	X	–
4 h	O	O	X	X	–
6 h	O	X	X	X	–
12 h	X	–	–	X	–
18 h	–	–	–	X	–
24 h	–	–	–	U	–

Notes: U = soluble in water; X = soluble in DMAc; O = insoluble in DMAc.

### 2.5. Effects of Drying Conditions

Variations in drying temperature and humidity were not found to significantly affect the studied membrane properties. Membranes cast at thicknesses of 250–300 μm were found to be suitably mechanically robust. It was difficult to maintain uniform thickness across the sheet for thicker membranes as the solution pooled in some areas. The actual thickness of the resulting membranes were dependent on drying conditions, with the DMAc solvent evaporating at different rates potentially influencing the mechanical structure of the membrane. 

The membranes were cast using a doctor blade to govern the thickness. As they were then dried at ambient pressures, the solution spread with time across the glass substrate. As such, the resulting dried membrane was significantly thinner than the cast thickness. The resulting membrane thickness was predominantly influenced by the concentration of the casting solution. Higher weight percentages of SPEEK polymer in DMAc resulted in thicker membranes. For mechanical stability and ease of handling, 30–50 µm was found to be most suitable. Using this method, 15%–25% casting concentrations SPEEK in DMAc were suitable to produce even and flexible membranes. 

The thickness and casting solution concentration had no discernable influence on the ion transport or water uptake properties of the membranes. These properties are expected to have an impact on the selectivity of the membranes due to changes in the microscopic pore structure [18]. However, in these initial studies, no significant trends were noted.

The drying temperature and humidity also had no notable influences on membrane swelling or ion transport. With higher humidity, longer times were required to remove all DMAc. It was noted that drying temperatures of 50–60 °C gave reasonable morphology without requiring long drying times.

### 2.6. Electrochemical Properties

The preparation conditions and properties of several membranes are summarized in Table 3. These membranes have electrochemical characteristics suitable for use in bioelectrical systems as well as appropriate water uptake to produce stable commercial membranes. 

**Table 3:** Summary of membrane properties when sulfonated at 40–60 °C for 6 h.

Temperature	Time	Cast Thickness	Casting Concentration	Actual Thickness	IEC	Water Uptake	Swelling	Potential Difference
°C	h	mm	%	mm	mequiv/g	%	%	V
40	6	0.2	10	0.0249	2.45	33.8	9.3	44.8
60	6	0.2	10	0.0245	2.25	62.1	17.7	105.4
60	6	0.3	15	0.0340	2.11	23.4	7.9	45
60	6	0.4	10	0.0360	2.25	47.8	14.8	61.7
60	6	0.2	25	0.0370	2.21	35.9	11.6	104.4
60	6	0.2	20	0.458	2.31	42.5	14.5	114.1
60	6	0.3	30	0.1097	2.27	34.5	14.7	61.7

In fuel cells and BESs, it is desirable for membranes to have high ionic conductivity while maintaining structural stability. From impedance spectroscopy, the resistance and conductivity of SPEEK membranes were found to be in the range of 10^−3^ S cm^−1^. Nafion^®^ 115 has conductivities in the range of 10^−2^ S cm^−1^ [14,26]. However, this lower conductivity is still feasible for applications in bioelectrical systems. Due to the significantly lower cost of SPEEK membrane materials and production, these membranes are highly attractive as an alternative to Nafion^®^ membranes.

## 3. Experimental Section

### 3.1. Materials

Poly(ether ether ketone) was provided ordered by Jida-Degussa High Performance Polymers (Changchun, China), and dried at 80 °C for 24 h before use. Sulfuric acid was supplied by Sinopharm Chemical Reagent Co. Ltd. (Shanghai, China).

Phenolphthalein was made up of 0.05 g phenolphthalein from Wenzhao Huaqiao Chemical Reagent Co., Ltd. (Wenzhou, China) in 50 mL ethanol. 

### 3.2. Sulfonation of PEEK

Concentrated sulfuric acid (>95%) was used as the sulfonating agent. This produces simple reactions and is known to produce polymers free from degradation or cross-linking reactions that can occur when using 100% H_2_SO_4_ or cholorosulfonic acid [3].

5 g of PEEK was gradually added to 50 mL sulfuric acid (95–98 wt %) in a three-necked round-bottomed flask. The flask was fitted with a mechanical stirrer and a condenser. The reaction mixture was heated in a water bath to the desired sulfonation temperature for the desired time while being vigorously stirred. The dissolved PEEK was a dark red, highly viscous solution. The PEEK was sulfonated at 40, 50, 60 and 70 °C for reaction times of 2, 3, 4, 5 and 6 h. 

The sulfonation reaction was terminated by precipitating the polymer in cold water. The precipitated SPEEK formed white noodle-like strands. These were soaked in water overnight and washed until the pH was neutral. The SPEEK polymer was dried at room temperature overnight and then in a vacuum oven at 60 °C for 24 h or longer as required to a consistent weight. 

### 3.3. Membrane Preparation

Membranes were prepared using the solvent evaporation technique. In a typical procedure, 3 g of SPEEK polymer was dissolved in DMAc and cast on a glass substrate. A doctor blade was used to control the nominal membrane thickness. Cast membranes were dried in a temperature and humidity controlled oven and then placed in a vacuum oven to ensure all residual solvent was removed. The membranes were immersed in water and peeled from the glass, then stored in deionized water before use.

Membranes were prepared with varying DMAc concentration, casting thickness, drying temperature and drying humidity. Membranes were cast with nominal (wet) thicknesses ranging from 200 to 400 μm, DMAc concentrations of 10%–30% and dried at temperatures of 40–80 °C. Drying humidity was originally varied from 50% to 90%, however, it was quickly determined that 60% was optimal for producing defect-free membranes, and was used for all subsequent experiments.

### 3.4. Characterization

#### 3.4.1. Ion Exchange Capacity

The ion exchange capacity provides information on the density of ionizable functional groups present in the membrane matrix, which are responsible for the charged nature of the membrane and thus membrane conductivity [19]. IEC is a measure of the number of counter ions exchangeable in SPEEK. IEC is defined as the milliequivalents of H^+^ per weight of the dry polymer.

The prepared SPEEK membranes were stored in 1 M HCl for 24 h to bring the sample into complete SPEEK-H^+^ form. Membranes were then rinsed in DI water and equilibrated in 1 M NaCl solutions for three days to completely exchange the cations and achieve SPEEK-Na^+^ form. 

The ion exchange capacity was measured using the back-titration method as described in several previous papers [16,18,20]. The NaCl solution was back titrated with 0.1 M NaOH using phenolphthalein as an indicator to determine the cations exchanged. The IEC was calculated as the ratio of total charge by dry weight of the membrane sample. 



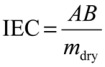
(2)


where *A* is the concentration of the NaOH solution used, *B* is the volume of NaOH solution consumed in the titration and *m*_dry_ is the dry weight of the membrane. 

#### 3.4.2. Water Uptake and Swelling

Soaked samples of the SPEEK membranes of approximately 1 cm × 5 cm were wiped and then weighed and measured to determine wet weight and wet length. These samples were then dried in a vacuum oven overnight and the dry weight and dry length determined. The water uptake and swelling ratios were determined by the following equations.

water uptake = [(wet weight − dry weight)/dry weight] × 100%
(3)

swelling = [(wet length − dry length)/dry length] × 100% × 100%
(4)

Membrane water content can also be defined as the number of moles of water per mole of ion exchange site; *λ* ≡ *H_2_O/SO_3_^−^*. This can also be equated as:

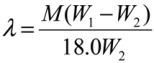
(5)
where *M* is the equivalent weight of the membrane, and *W*_1_ (g) and *W*_2_ (g) are the weights of a membrane piece before and after drying *in vacuo*.

#### 3.4.3. NMR

The NMR spectra of SPEEK samples sulfonated at 50 °C-2 h; 50 °C-6 h, 60 °C-2 h and 60 °C-6 h were tested at Xiamen University, China using a Bruker 400 Avance II NMR spectrometer. SPEEK samples were dissolved in DMSO-d6 at concentrations of 2–5 wt %. The chemical shift of tetramethylsilane (TMS) was used as the internal standard reference.

Non-sulfonated PEEK is insoluble in any solvent except for strong acids, hence no 1H-NMR spectra could be recorded for pure PEEK.

#### 3.4.4. Potential Difference

When a membrane separates electrolyte solutions of unequal concentration, an electrical potential difference develops across the membrane [22]. The developed potential across the membrane in a two-compartment cell was recorded with a digital multimeter. The membrane was separated by 0.01 mol dm^−3^ NaCl and 0.05 mol dm^−3^ NaCl.

The transport number, *t*_+_, can be calculated using the following equation:

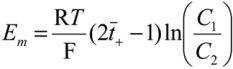
(6)
where R is the gas constant, F is the Faraday constant, *T* is the absolute temperature, *C*_1_ and *C*_2_ are the concentration of electrolyte solutions in the testing cell. 

The transport number, or transference number, is the ratio of the current carried by a certain ionic species through a cross section of the electrolyte over the total current. It is dependent on the mobilities of all the ions in the electrolytic solution, on the concentrations of the ions and on the temperature of the solution [28]. The ion transport number indicates the different contribution of ions to the electric current due to different electrical mobility. This gives an indication as to the rate of ion permeation and selectivity of the membrane.

When the membrane transport number decreases, this indicates that the membrane is less permselective.

The transverse proton conductivity has been shown to increase from 4.1 to 9.3 × 10^−3^ S cm^−1^ with an increase in the degree of sulfonation from 0.59 to 0.93 [15]. 

## 4. Conclusions

In this work, PEEK was sulfonated by sulfuric acid at various sulfonation conditions to introduce negatively charged groups into the polymer chains. A series of SPEEK membranes were produced using a range of reaction time and temperature to compare resultant membrane performance and the effects on the degree of sulfonation. A series of casting conditions involving solvent casting concentration and thickness was also compared to determine optimal membrane casting conditions. From the results of this novel systematic evaluation of the performance of each membrane produced in this series, ideal preparation conditions for PEEK sulfonation and membrane casting were established. This work will be used in further developing a uniquely suitable cation exchange membrane for specific wastewater BESs with the aim of extracting chemicals.

The influence of temperature was noted to be more significant on the sulfonation degree than reaction time. The DS determined using the titration method and the ^1^H-NMR method were found to be consistent, representing accurate quantification of this variable. The most appropriate sulfonation conditions were noted as being 60 °C and 6 h to produce high ion exchange capacity membranes with appropriate water uptake. 

The membrane thickness was predominantly influenced by the concentration of the casting solution. Higher weight percentages of SPEEK in DMAc resulted in thicker membranes. For mechanical stability and ease of handling, membranes in the thickness range of 30–50 µm was most suitable. 15%–25% concentrations should be used to produce these membranes. While the drying conditions did not have an apparent impact on the measured parameters in this study, further investigation will look at the influence of these conditions on controlling porosity and selectivity.

To obtain the most desirable membrane properties of ion exchange capacity and high cation conductivity, while still maintaining thermal and mechanical stability, the sulfonation and preparation conditions for SPEEK membranes has be optimized with large-scale production in mind. The findings from this study will lead to a better understanding of the effect of the polymer preparation conditions on SPEEK membrane performance. These membranes will be implemented in specific BESs for the extraction of chemicals from industrial wastewater. Future work will involve testing the optimized SPEEK membrane in such BESs to evaluate their performance and the lifetime of such materials under expected caustic conditions. A cost analysis of these membranes for future commercial production will also be determined.
